# The Relationship Between Technetium-99m-Methoxyisobutyl Isonitrile Parathyroid Scintigraphy and Hormonal and Biochemical Markers in Suspicion of Primary Hyperparathyroidism

**DOI:** 10.4274/Mirt.21931

**Published:** 2013-04-05

**Authors:** Güler Silov, Ayşegül Özdal, Zeynep Erdoğan, Özgül Turhal, Hatice Karaman

**Affiliations:** 1 Kayseri Training and Research Hospital, Department of Nuclear Medicine, Kayseri, Turkey; 2 Kayseri Training and Research Hospital, Department of Pathology, Kayseri, Turkey

**Keywords:** Technetium-99m sestamibi, primary hyperparathyroidism, parathyroid hormone, calcium, phosphorus

## Abstract

**Objective:** Technetium-99m-methoxyisobutyl isonitrile (Tc-99m MIBI) has been widely used to evaluate hyperfunctioning autonomous parathyroid glands in patients with elevated intact parathyroid hormone (iPTH) and/or calcium (Ca) level. The aim of this study was to evaluate the relationship between Tc-99m MIBI parathyroid scintigraphy and hormonal and biochemical markers in suspicion of primary hyperparathyroidism (PHPT).

**Material and Methods:** Dual-phase Tc-99m MIBI parathyroid scintigraphy and total serum iPTH, Ca, phosphorus (P) and albumin measurements were performed in 60 patients (52 females, 8 males; mean age, 59.38±12.51 years; range, 34 to 86 years) with suspicion of PHPT.

**Results:** The iPTH median level was 160.3 pg/mL (47.8 to 782.6). Thirty-five of the patients had surgical resection of hyperfunctioning parathyroid glands. Of the 35 patients, parathyroid gland pathology was detected in 30 patients using scintigraphic examination. Tc-99m MIBI parathyroid scintigraphy was negative in 30 patients. The iPTH, Ca and P levels were significantly different between in the Tc-99m MIBI positive group and the negative group, respectively: For iPTH, 202.1 (47.8-782.6) pg/mL versus 111.6 (80.1-373) pg/mL; p<0.001. For Ca, 11.7±1.15 mg/dL versus 10.3±1.05 mg/dL; p<0.001 and for P levels, 2.46±0.62 mg/dL versus 3.40±0.70 mg/dL; p<0.001). There was no significant difference in serum albumin levels between the MIBI positive and MIBI negative groups (4.25±0.27 g/dL versus 4.25±0.41 g/dL; p>0.05). Tc-99m MIBI parathyroid scintigraphy showed good correlation with iPTH level and histopathological diagnosis. Sensitivity and specificity was found 83.3% and 76.7%, respectively at the level of iPTH>147.7pg/mL.

**Conclusion:** Tc-99m MIBI parathyroid scintigraphy is most likely to produce identification and localization of a parathyroid adenoma when both iPTH and Ca are elevated as well as decreased P levels.

**Conflict of interest:**None declared.

## INTRODUCTION

Primary hyperparathyroidism (PHPT) is relatively a common endocrine disorder usually eventuate due to autonomous overproduction of parathyroid hormone (1). The typical biologic profile of the disease is associated with elevated serum total and/or ionized calcium (Ca) and intact parathyroid hormone (iPTH) concentrations. Normal serum Ca concentration alongwith elevated iPTH levels may sometimes be seen in early phases of PHPT (2). PHPT is generally associated with an increased risk of osteoporosis, hypertension and cardiovascular morbidity (3), as well as impairment in cognitive functions and in quality of life (4). Surgery, as the main curative treatment, may improve some of these complications, particularly those related to bone metabolism irregularities (5). As the underlying reason is solitary adenoma in approximately 90 percent of patients with PHPT, preoperative localization of parathyroid adenoma is crucial, particularly when a targeted surgical approach (minimally invasive parathyroidectomy) is planned (6).

Technetium-99m-methoxyisobutyl isonitrile (Tc-99m MIBI) parathyroid scintigraphy, using a dual-phase procedure, has been widely used in preoperative localization of hyperfunctioning parathyroid glands in patients with with hyperparathyroidism (HPT). It is useful for patients with PHPT, especially for those with solitary adenomas (7). The uptake of Tc-99m MIBI may be influenced by a variety of biological factors. These include the size of the adenoma, the cell type, the P-glycoprotein expression, and the mitochondrial structure (8). When p-glycoprotein expression increased, the efflux of Tc-99m MIBI from the tumor can hinder the visualization of tumors. Serum Ca levels may modulate radiotracer kinetics by influencing the membrane potential (8). These processes jointly establish the basic mechanism of this imaging method.

The iPTH level reflects the functional status of the parathyroid glands and has become one of the most important diagnostic tests for HPT. The iPTH level has been shown to be higher than normal in more than 90% of patients with HPT (10).

The aim of the present retrospective study was to assess the relationship between Tc-99m MIBI parathyroid scintigraphy and serum iPTH, Ca, phosphorus (P) and albumin levels in suspicion of PHPT. 

## MATERIALS AND METHODS

**Patients**

Between February 2010 and January 2012, 60 patients were referred to our Nuclear Medicine Department for Tc-99m MIBI parathyroid scintigraphy due to serum Ca and/or iPTH abnormalities consistent with HPT. The patients with severe kidney failure and/or those suspected to have secondary or tertiary HPT were excluded. 

**Methods**

Serum iPTH (reference range: 11.0-79.5 pg/mL) was measured using commercially available immunometric assay, Ca (reference range; 8.4-10.6 mg/dL), P (reference range; 2.5-4.7 mg/dL) and albumin (reference range; 3.5-5.0 g/dL) were measured using spectrophotometric analysis.

**Dual Phase Tc-99m MIBI Parathyroid Scintigraphy**

Patients were injected intravenously with 555 MBq (range, 520-572) of Tc-99m MIBI. The image acquisitions were performed on a double-head gamma-camera equipped with LEHR collimator (GE Infinia, USA). Early and delayed neck planar images were obtained 10 and 90 and 180 min after injection. Anterior neck images were acquired for 10 min, in a 128x128 matrix. The camera was set for a 140 keV photo peak with a 20% window. Single photon emission computerized tomography (SPECT) acquisition was performed immediately after the first delayed planar image. The SPECT volume session included the neck and thorax. A 128x128 matrix was used and images were obtained with 60 projections at 30 sec over 360 degrees. Slice thickness was 4.42 mm. Data from SPECT were reconstructed using a 3-dimensional iterative algorithm (ordered-subsets expectation maximization with 2 iterations and 10 subsets). Images were smoothed with a 3-dimensional spatial Hann filter. Data from SPECT were analyzed on an e-soft workstation, providing transaxial, sagittal, and coronal slices of SPECT. The interpretation of Tc-99m MIBI parathyroid scintigraphy was performed in consensus by 2 experienced nuclear medicine specialist physicians. The image findings were binary scored as positive or negative. Scintigraphy was positive when focal tracer retention in the neck or in the mediastinum was clearly evidenced on planar and/or SPECT images. Scintigraphy was reported as negative when focal uptake in the neck or in the mediastinum was evidenced neither on early nor on delayed planar and/or SPECT images.

According to the overall evaluation, including Tc-99m MIBI parathyroid scintigraphy, patients underwent surgery or were just followed-up. Histopathologic examination was done. Clinical chemistry profile data were expressed as mean ±SD. Endocrinological data were expressed as median, minimum and maximum values.

Statistical analysis was performed using Student’s t test, Mann-Whitney U tests, and Paired-Samples T test. In addition, ROC curve was used for the statistical evaluation of the cut-off values. The level of statistical significance was set at 0.05.

## RESULTS

**Patient Characteristics**

The study group included 60 patients with a mean age of 59.38±12.51 years (range; 34-86). There were 52 women (86%) and 8 men (14%). The median serum iPTH level was 160.3 pg/mL (range 47.8-782.6 pg/mL), the mean total serum Ca level was 11.02±1.29 mg/dL (range 9-15.3 mg/dL) and the mean serum P level was 2.92±0.81 mg/dL (range 1.5-5 mg/dL).

**Tc-99m MIBI Data Results**

**Figure 1:**
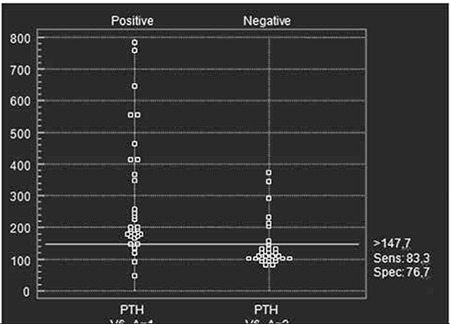
Sensitivity and specificity of iPTH at the level of 147.7 pgml

Overall, 30 patients were MIBI positive (Table 1) and 30 patients were MIBI negative (Table 2). Tc-99m MIBI parathyroid scintigraphy showed good correlation with histopathological diagnosis. Among the 30 MIBI positive patients, 30 subsequently underwent parathyroidectomy and were proved to be true positives by histopathologic examination. Five of the 30 patients with MIBI negative studies underwent surgery and histopathologic reports were available. The iPTH levels were significantly higher in the patients with MIBI positive scans than in those who were MIBI negative 202.1 (47.8-782.6) pg/mL versus 111.6 (80.1- 373) pg/mL; p<0.001) (Table 3). There was significant difference in serum Ca levels between the MIBI positive and MIBI negative groups (11.7±1.15 mg/dL versus 10.3±1.05 mg/dL; p<0.001). Serum Ca and iPTH concentrations were correlated in the whole series of patients (r=0.424, p<0.001). There was significant difference in serum P levels between the MIBI positive and MIBI negative groups (2.46±0.62 mg/dL versus 3.40±0.70 mg/dL; p<0.001). There was no significant difference in serum albumin levels between the MIBI positive and MIBI negative groups (4.25±0.27 g/dL versus 4.25±0.41 g/dL; p>0.05). Serum P and iPTH concentrations were correlated in the whole series of patients (r=-0.450, p<0.001). MIBI was positive in 30 of 60 (50%) patients; 54 of 60 (90%) patients with iPTH levels greater than 100 pg/mL and 34 of 60 (56%) patients with iPTH values greater than 147 pg/mL. Sensitivity and specificity was found as 83.3% (CI;65.3-94.3), 76.7 % (CI;57.7-90.0) respectively at the level of iPTH>147.7 pg/mL (Figure 1).

## DISCUSSION

In recent years, Tc-99m MIBI parathyroid scintigraphy has successfully been used in the localization of parathyroid glands prior to surgical parathyroidectomy in patients with PHPT. This technique guides for identification of the gland which should be removed so as to diminish the risk of recurrence (10).

In our study, 30 MIBI positive patients of a total of 60 patients underwent parathyroidectomy and had parathyroid adenomas as proved by histopathologic analyses.

In 5 of 30 patients who were MIBI negative, the results of Tc-99m MIBI parathyroid scintigraphy were categorized as false negative (FN). Early washout was seen two of 5 FN patients. But in the rest of these patients, we did not observe significant focal uptake on early planar images. This suggests that fast washout might not have the only main impact on FN studies (11). According to overall initial and follow-up evaluations, remaining 25 patients were clinically accepted as true negative patients and did not undergo surgery. The recognition of HPT is founded on biochemical and hormonal markers. A hypercalcemia founded on repetetive serum Ca measurements and is accompanied by an elevated or inappropriately high-normal iPTH level is indicative of HPT. Of the 29 patients who had high level of iPTH and positive scan results, only 2 patients had plasma Ca level lower than upper limit of reference range. This is described as normocalcaemic HPT and may be an suggestion of early diagnosis of the disease. On contrary, of the 25 patient who had high level of iPTH and negative scan result, 19 had plasma Ca levels lower than upper limit of the reference range. In these patients, the diagnosis of the PHPT and request for Tc-99m MIBI parathyroid scintigraphy seem to be unsuitable. One can also suppose that some of the patients with blood chemistry and hormonal irregularity may have disorders other than PHPT such as vitamin D deficiency or familial benign hypocalciuric hypercalcemia (12).

In our study, only one patient whose scan finding was positive, had a normal iPTH and this was at the median limit of reference range in our instutition. Although it is to be considered that the plasma Ca of the same patient was in the hypercalcaemia range. 

Most studies published to date agree that MIBI positive scintigraphy may have a close relationship with parathyroid gland function (13). However absence of any statistically significant difference in serum Ca levels between MIBI positive group and MIBI negative group has previously been reported (14). Controversially some other studies have postulated a statistically significant positive relation between MIBI positive scintigraphy and Ca levels (10,13,15). In accordance with the previous studies (10,13,15), our data shows that the serum Ca and iPTH levels have a positive relation with positive results of Tc-99m MIBI parathyroid scintigraphy as a consequence of parathyroid gland function (13). We also found that the positive rates were higher in the groups with higher iPTH levels. Sensitivity and specificity was highest at the level of iPTH>147.7pg/mL. 

In addition, in our study there was a negative relation between MIBI positive scintigraphy and P levels. Mean P levels was found in the normal reference range in MIBI negative group, at the bottom of the reference range in MIBI positive group. The reverse relation between the changes in serum P levels and MIBI uptake has been found only in a few studies previously (9,15). The main possible cause for not investigating this topic is that P does not play a role in cellular uptake and wash out mechanisms of Tc-99m MIBI. It was estimated that, the main cause that underlies this contrary relation between serum P level and MIBI positivity is due to the opposed relation between Ca and P (9). In fact these alterations and correlations in concentrations of analytes like iPTH, Ca and P those are observed in HPT are indicators of increased metabolism of pathologic parathyroid glands and we can conclude that Tc-99m MIBI parathyroid scintigraphy should be accepted as a functional testing for PHPT.

In conclusion, Tc-99m MIBI parathyroid scintigraphy is most likely to produce identification and localization of a parathyroid adenoma when both iPTH and Ca are elevated as well as decreased P levels.

## Figures and Tables

**Table 1:**
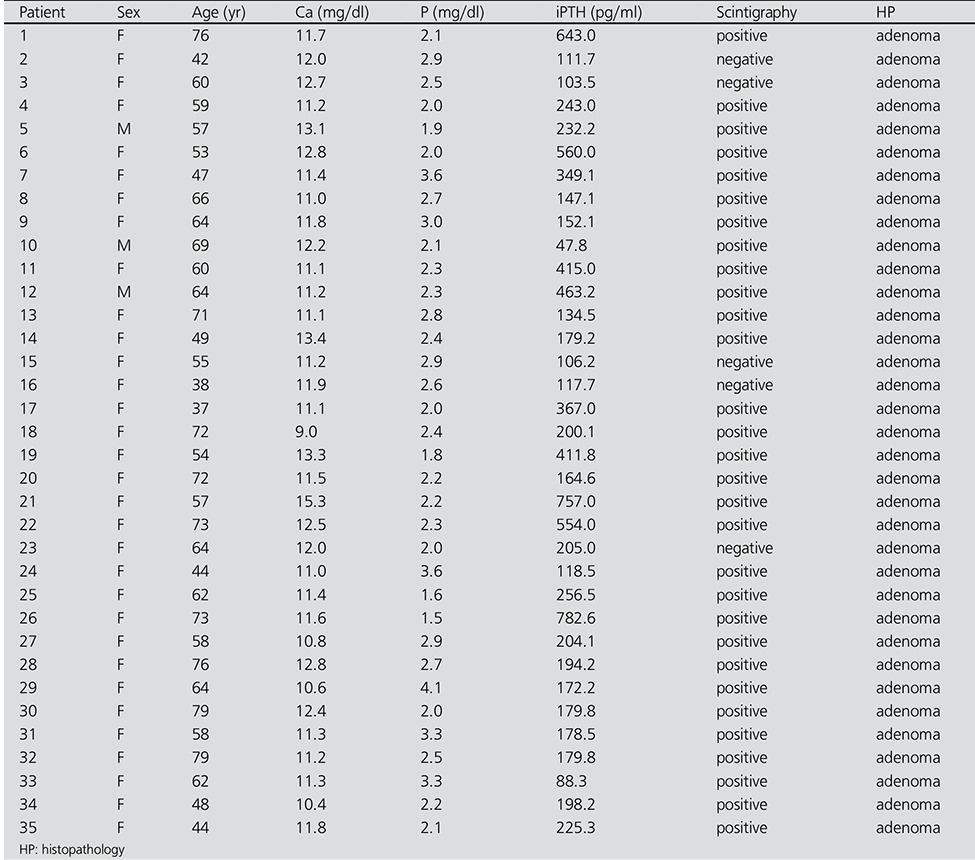
Characteristics of Patients with PHPT

**Table 2:**
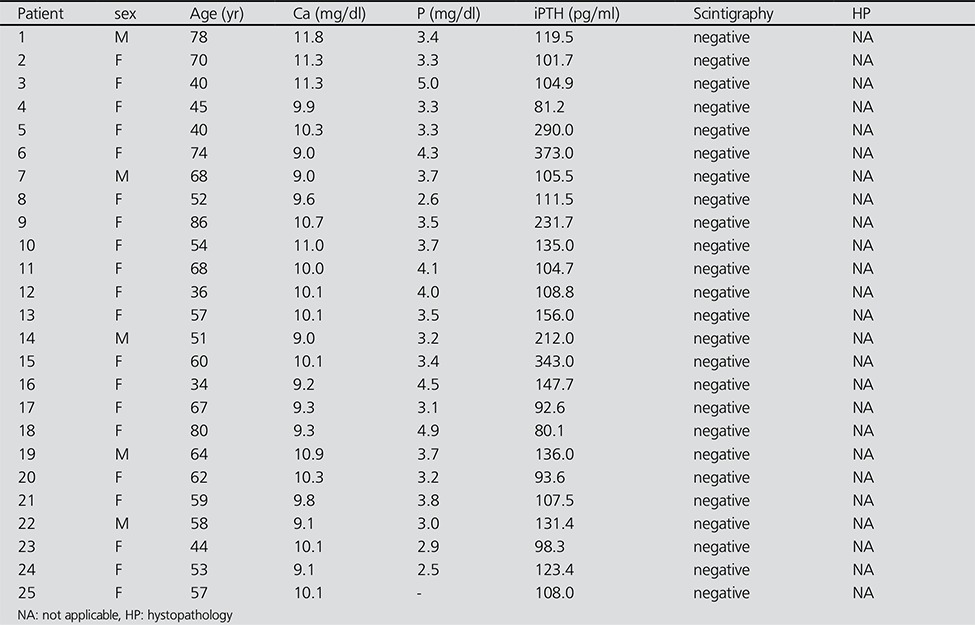
Characteristics of adenoma negative patients

**Table 3:**
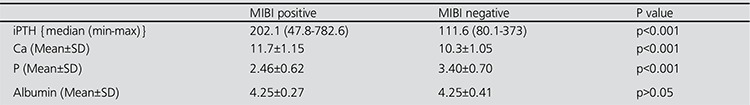
Statistical Data of Intact Parathyroid Hormone (pg/ml), Ca (mg/dl), P(mg/dl), Albumin (g/dl)
